# The Principles of Surgery

**Published:** 1837-04

**Authors:** 


ART. IX.—*The Principles of Surgery.* By JAMES SYME, F.R.S. E., Professor of Clinical Surgery in the University of Edinburgh. *Second Edition.—Edinburgh,* 1837. 8vo. pp. 460.

MR. SYME is favorably known to the profession by his Treatise on "Excision of the Joints," and his numerous communications in the Edinburgh Medical and Surgical Journal. Those who are acquainted with the Edinburgh schools of medicine are well aware of his unwearying zeal and energy in the pursuit of his profession. Previous to his present appointment of professor of clinical surgery, he established a considerable hospital at his own expense, and taught clinical surgery to one of the most numerous classes frequenting private lecture-rooms; so that, both as a practical surgeon and as a teacher, he has been long acquiring that kind of information, and training his mind to those pursuits, which are most fitted to qualify the individual for the production of an elementary work on Surgery. Lecturers are, of all others, the most proper persons to write elementary treatises, from their habits of going over, year after year, all the details of every department of the branch they profess, and of presenting them in their most simple form to those commencing the studies of their calling. Indeed, the German professors appear almost to consider it to be a part of their duty to write text-books for their pupils; and in this way many admirable works of more than local interest have originated. Many books of this kind have also originated from the Edinburgh schools, as the treatises of Liston, Alison, Christison, Hamilton, M'Intosh, Campbell, Syme, Craigie, and others, on the respective subjects which they profess, abundantly testify. The present work of Mr. Syme has reached a second edition: its first appearance was in two volumes, with ample margins and most readable print; but Mr. Syme, after the more economical fashion of the last few years, has republished it in one closely printed volume, with smaller type, at a little more than half the price of its predecessor; although he has revised the old matter, and added much that is new. The critic's task with books of this kind is one of some difficulty, especially if his aim is practical usefulness, as well as just criticism. It is out of the question to analyze matter that should have been compressed into the smallest compass; nor will mere extracts, isolated bits, like the separate stones of a building, produce any interest, or give a very clear notion of the author's design: it is delicate to criticise omissions where much must be, of necessity, omitted, or to object to a want of originality where originality may be carried too far. Perhaps the most feasible plan is merely to give a general opinion, such as every reflective reader forms after perusing a book.

Mr. Syme's peculiar excellence appears to us to depend on his minute observation, on his power of describing clearly those appearances which have fallen under his own inspection, and on his aptness at methodical arrangement. He is less capable of clearly stating great principles and leading truths. For instance: his description and classification of ulcers is clear, intelligible, and graphic; but his chapter on inflammation gives a less decided impression of his having thought out the subject for himself, or even of his having made himself so thoroughly the master of the opinions of others as to reproduce them with that degree of originality which would prove that he had completely digested them. This criticism however applies to a very small part of the book, which we hold to be a highly useful and creditable performance; one that we should unhesitatingly recommend to young men in need of elementary works, and one from which even those who have been long practising their profession may gain serviceable information. The minor operations of surgery are described with as much minuteness as the more severe; and all are easily intelligible. The following description of excision of the joints is a fair specimen of Mr. Syme's method of compressing much into a little space, without sacrificing clearness. By those who have not seen his work, it will be read with interest.

*Excision of the Joints.* "Amputation has until lately been regarded as almost the only means of relief from carious joints. But it is now ascertained by experience, that the limb may be saved by cutting out the articulation. The softened, discoloured, and ulcerated integuments, the thickened and indurated cellular substance, and the gelatinous synovial membrane, are found to afford no serious obstacle to recovery, provided the whole of the bones, so far as they are actually carious, are taken away. The operation requisite for this purpose, though severe, is not more dangerous than amputation, because the joint, previous to its performance, has been opened by the disease; the whole of the articulating tissues which are apt to suffer violent inflammation when irritated are either destroyed or removed; the great blood-vessels and nerves are not interfered with, and the patient is not subjected to the shock which is caused by taking off a limb." (P. 211.)

"*Shoulder-Joint.* Different methods have been followed in cutting out the shoulder-joint, but it will be sufficient to describe the one which appears to be the most convenient. The patient being seated on a chair, and properly supported, the surgeon introduces a straight, sharp-pointed knife under the acromion, thrusts it down to the head of the humerus, and then cuts perpendicularly, close upon the bone, nearly as far as the attachment of the deltoid. He next carries the knife backwards and upwards from the inferior extremity of the first incision, so as to divide the external part of the deltoid. And, having thus formed a flap, he dissects it from the subjacent parts, so as to expose the articulation. In order to detach the head of the humerus, which is of course his first object, he cuts transversely into the joint, introduces the forefinger of his left hand, and, using it as a guide for the knife, separates the attachments of the muscles, which are inserted into the greater and smaller tuberosities. The arm being then drawn across the breast, the head of the bone protrudes the wound, and, being grasped in the hand, may be readily sawn off. The glenoid cavity should next be examined, and taken away as far as seems necessary, which is easily done with the cutting pliers. The whole of the surface covered with cartilage should always be removed, and in general this will be sufficient; but sometimes the caries extends farther into the bone, and in this case must be carefully followed out by the pliers or gouge.

"The only artery cut during the operation that requires a ligature, is the posterior circumflex. The edges of the wound should be stitched together, and, some light dressing having been applied, the arm ought to be supported by a spica bandage and sling.

"The patient need not be confined to bed beyond a day or two, or so long as the fever excited by the operation continues; and, when the wound begins to heal, he must assiduously exercise the limb, to prevent it from becoming stiff.

"*Elbow-Joint.* The best mode of performing the excision of the elbow-joint is that which was originally contrived and practised by Moreau. The patient should lie with his face downwards, so as to present the posterior surface of the joint. The surgeon, using the same kind of knife which was recommended for the former operation, makes a transverse incision into the joint, close above the olecranon, and extending from the inner edge of the process to the external tuberosity of the humerus. It is necessary, in doing this, to be careful to avoid the ulnar nerve, which lies close upon the inner side of the olecranon; and the safest plan is to thrust down the knife perpendicularly into the joint, with its back directed towards the nerve. At each extremity of the transverse cut thus made, the surgeon next makes an incision about an inch and a half long, both upwards and downwards, in the long direction of the limb, so as to form two square flaps, and give the form of the wound a resemblance to the letter H. These flaps being detached from the parts below them, the olecranon may be easily removed by the saw or pliers, after which no difficulty will be experienced in cutting the lateral ligaments of the joint, protruding the extremity of the humerus, and sawing it off through the tuberosities. Lower than this would not be sufficient for removing the whole of the cartilaginous surface, and the caries very rarely extends higher up. The head of the radius may next be cut away with the pliers; and then nothing remains to be done but the separation of the portion of the sigmoid cavity of the ulna that was left after the removal of the olecranon, which may now be readily effected by the pliers. It might be thought better to take away all of the ulna that required excision at once, but the attachment of the brachiæus internus to the coronoid process renders this very difficult, especially if it is attempted before the free space afforded by the removal of the other bones is obtained. After the olecranon and the extremities of the humerus and radius are detached, it is easy to cut out with the pliers any more of the ulna that may be required.

"It is seldom necessary to tie any arteries; but, if a disposition to bleed should be observed when the operation is finished, the vessels ought to be sought for and secured, however small; as the hemorrhage, when allowed to continue, produces very disagreeable effects by distending the wound, separating its edges, and causing great irritation. The wound should be closed with stitches of the interrupted suture, and then a long bandage must be applied in the figure of 8 to support the limb, which should be bent at a right angle, and to prevent the ends of the bones from moving or pressing injuriously on the soft parts. Rigid cases of iron or wood have been proposed for this purpose, but they are found to be in all respects less convenient than the means just mentioned. The patient, after the first two or three days, will find himself most comfortable in the erect posture; and, when the inflammatory tension consequent upon the operation begins to subside, he should gently but diligently exercise the limb, so as to preserve the mobility of the elbow." (P. 212.)

